# The shape of lipsmacking: socio-emotional regulation in bearded capuchin monkeys (*Sapajus libidinosus*)

**DOI:** 10.1017/ehs.2023.10

**Published:** 2023-05-12

**Authors:** Natalia Albuquerque, Carine Savalli, Marina Belli, Ana Clara Varella, Beatriz Felício, Juliana França, Patrícia Izar

**Affiliations:** 1 Institute of Psychology, University of São Paulo, São Paulo, Brazil; 2 Federal University of São Paulo, São Paulo, Brazil

**Keywords:** Emotions, emotional regulation, lipsmacking, primates, *Sapajus libidinosus*

## Abstract

Capuchin monkeys have rich social relationships and from very young ages they participate in complex interactions with members of their group. Lipsmacking behaviour, which involves at least two individuals in socially mediated interactions, may tell about processes that maintain, accentuate or attenuate emotional exchanges in monkeys. Lipsmacking is a facial expression associated with the establishment and maintenance of affiliative interactions, following under the ‘emotional regulation’ umbrella, which accounts for the ability to manage behavioural responses. We investigated behaviours related to the emitter and to the receiver (infant) of lipsmacking to answer the question of how lipsmacking occurs. In capuchin monkeys, lipsmacking has been previously understood solely as a face-to-face interaction. Our data show that emitters are engaged with infants, looking longer towards their face and seeking eye contact during the display. However, receivers spend most of the time looking away from the emitter and stay in no contact for nearly half of the time. From naturalistic observations of wild infant capuchin monkeys from Brazil we found that lipsmacking is not restricted to mutual gaze, meaning there are other mechanisms in place than previously known. Our results open paths to new insights about the evolution of socio-emotional displays in primates.

## Graphical abstract

**Figure:**
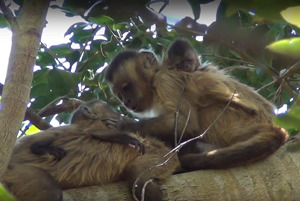

**Social media summary**: Lipsmacking in capuchin monkeys is not restricted to mutual gaze and face-to-face interactions.

## 1. Introduction

Emotions channel individual responses in certain directions in accordance to how animals relate to, perceive and react to their environment (Albuquerque et al., 2018) and will provide individuals with a toolkit to interact with the world, which consists of emotional regulation mechanisms. According to Davidson et al. (2000), emotional regulation consists of any processes that maintain, attenuate or accentuate emotional reactions. In fact, it is a multifaceted process responsible for inhibiting or starting responses triggered by various stimuli (Horato et al., 2022). These mechanisms involve perception, recognition, inferential skills, responsiveness, experience and expression. The study of emotional regulation allows examination of how individuals adjust to their social and physical world, encompassing how animals react to a variety of stimuli (Maestripieri, 1999). Emotional processes consist of responses to certain stimuli with the activation of adaptations to the physical and social environment (Evers et al., 2014). Emotional experiences, fundamentally based on two affective reactivity dimensions (one aversive, one rewarding), will result in adaptive behaviours and behavioural patterns. Cervone and Pervin (2013) discuss that these behavioural tendencies grounded on emotional experiences function as mediators of the influences of external means and can be seen from early ages.

Emotions play a fundamental role in the lives of animals, especially for species that constitute cohesive social systems (Parr et al., 2000). According to Gross (1998), emotional experiences will have a two-fold role: (a) at the individual level, since they allow an animal to assess and react to a variety of stimuli in an appropriate way; and (b) at a social level, since they allow the transmission of ecologically relevant information to other individuals in the same group. Even though the study of expression and perception of emotion in non-human animals is becoming more common, with important evidence in monkeys (Ghazanfar & Logothetis, 2003), horses (Smith et al., 2016; Proops et al., 2018; Nakamura et al., 2019), dogs (Müller et al., 2015; Albuquerque et al., 2016, 2021; Albuquerque & Resende, 2023) and cats (Galvan & Vonk, 2016), among others, little is known about how individuals deal with physical and social emotion-eliciting stimuli.

Moreover, emotional processes are central to the exhibition of affiliative behaviours and to the regulation of social interactions (e.g. Boissy et al., 2007). In this study we will look at a behaviour that is a very good candidate for the study of emotional regulation: the lipsmacking behaviour, a rapid closing and opening of the mouth and lips (Fedurek et al., 2015). Lipsmacking is a facial expression that is related to the regulation of affiliative behaviours, both when performed and received by capuchin monkeys. In fact, De Marco and Visalberghi (2007) discuss that lipsmacking is the first display to be exhibited by young capuchins. According to these authors, lipsmacking has an affiliative function, sending a positive message and promoting affiliative interactions. Lipsmacking is a multimodal signal, as it integrates visual and acoustic perceptual domains. However, evidence has shown that the visual element is sufficient to elicit reciprocation (Fedurek et al., 2015).

According to Horato et al. (2022), emotional regulation refers to the ability to manage our behavioural responses when facing everyday situations. Lipsmacking seems to account for that as it is one of the most versatile displays in non-human primates in terms of the context of production, from infant caring to subordination, even within the same species (Gallo et al., 2022). Letting others know what an individual wants is especially important in initiating and maintaining cooperative or friendly interactions involving close physical proximity. As a consequence, animals such as chimpanzees have evolved behaviours to signal benign attitudes prior to or during a cooperative interaction (Fedurek et al., 2015). Fedurek et al. (2015) suggest that lipsmacking in chimpanzees functions to maintain and prolong grooming bouts, as well as to facilitate reciprocity during grooming. According to the authors, lipsmacking in chimpanzees may serve as a coordinator and regulator of other affiliative behaviours, especially grooming, which may increase in frequency and intensity depending on the area of the body to which grooming is directed and visual contact. Lipsmacking is a rhythmic facial expression that is an affiliative behaviour directed towards another individual and is one of the behaviours seen within the repertoire of face-to-face interactions (Pereira et al., 2021). In non-human primates, lipsmacking appeases the recipient of the behaviour and facilitates affiliation (Evers et al., 2014). Some studies have been assessing its frequency, duration and inter-individual variability, as well as the tuning process throughout ontogeny (Bergman, 2013). Lipsmacking is related to the regulatory mechanisms of the infant (Bergman, 2013) and in mother–infant interactions this display may be presented in an exaggerated way in combination with mouth-to-mouth contact (Ferrari et al., 2009).

However, we are far from a full comprehension of its function, especially since this display can vary across species, individuals and contexts. Therefore, further studies are needed to address issues such as: is lipsmacking strictly a face-to-face interaction? Is it exclusively a mother–infant behaviour? Is lipsmacking linked to other aspects of capuchin monkeys’ social development? In this study, we looked at individual phenomena, with a focus on the characteristics of the shape of lipsmacking (e.g. direction, duration, frequency).

Intrinsic variables, such as age and sex, as well as extrinsic variables (e.g. environmental demands) might influence the development and exhibition of a variety of behaviours. One of the potential predictor variables is the quality of the relationship between mother and offspring (Verderane et al., 2020). Verderane et al. (2020) investigated capuchin monkeys (*Sapajus libidinosus*) in Brazil and to assess the quality of these mother–offspring relationships, they measured physical contact, tactile stimulation and face-to-face behaviours. They found that lipsmacking was a face-to-face behaviour, which relates to spatial proximity and physical contact and allows social co-regulation.

Studying lipsmacking may provide a better understanding of socio-emotional regulation. For that matter, one needs to investigate the underlying mechanisms that relate to how this behaviour occurs and how this sort of interaction is regulated. Aspects such as the receiver's gaze direction during lipsmacking, the emitter's gaze direction, the active search of the emitter for a face-to-face interaction with the infant, and physical contact between receiver and emitter during the exhibition of the display might manage the occurrence of the behaviour. Here, we do not look at the modification of behaviour. Instead, we take a step back to address prior questions of how lipsmacking, a facial expression known to be linked to emotional expression and socio-emotional regulation, occurs.

One of the most interesting features of capuchin monkeys is that, as platyrrhines, they raise their offspring on their back, while other primate species (catarrhine) carry and transport theirs under their belly. This results in less visual contact with the mother and greater possibilities for visual contact with other individuals. Affiliative behaviours, such as grooming, are important for the maintenance of social interactions in several species, including capuchin monkeys (e.g. Tiddi et al., 2010, 2011). According to Thompson and Cords (2019), grooming has different functions when performed with the mother and with other individuals. We expect that the same might be true for other behaviours, such as lipsmacking, from its informational, regulatory and affiliative promoting aspects.

The purpose of this study was to expand on our understanding of lipsmacking as a regulator of socially and emotionally mediated interactions, such as those involving the display. We conducted in-depth investigations of wild infant capuchin monkeys (*Sapajus libidinosus*) of Fazenda Boa Vista (Piaui, Brazil) concerning the lipsmacking behaviour (Pereira et al., 2021) to generate data on its structure. We investigated the structural aspects of this behaviour. Our first aim was to examine whether the duration of lipsmacking would be affected by intrinsic variables. We also tested the hypothesis that lipsmacking is a face-to-face behaviour. We predicted that: (a) the time infants spent looking at this specific facial display would be higher than looking at other parts of the emitter's body; (b) the time infants spent in physical contact with the individual who is displaying the expression would be greater than in no contact; (c) the time that emitters spent looking at the infants’ face would be higher than looking at other body parts; and (d) the time that emitters spent actively seeking the face of the baby would be greater than not seeking. Our second hypothesis was that lipsmacking is a behaviour that occurs between mother and infant, with the prediction that (e) occurrence of the display would be greater with the mother than with non-mother individuals. We also looked at what we are calling ‘associated facial expressions’, which are expressions (e.g. scalp lifting, tongue protrusion) displayed concurrently to lipsmacking. We aimed to test the hypothesis that these associated expressions play a role in the occurrence of lipsmacking and the ‘lipsmacking interaction’ between emitter and receiver, since redundant emotional information may boost discriminatory processes, with the prediction that (f) when these expressions occur lipsmacking displays are longer.

## 2. Methods

### 2.1. Ethical approval

This study consisted exclusively of naturalistic observations. Video recordings were made by two experienced, well-trained field assistants, to which the animals were highly habituated. There was no handling or manipulation of the analysed individuals. None of the monkeys were subjected to any sort of stressful or uncomfortable situation. The procedures of this study were approved by the Ethics Committee for Animal Research of the Institute of Psychology, University of São Paulo, CEUA no. 6870180216.

### 2.2. Subjects

We analysed the behaviour of 10 wild bearded infant capuchin monkeys. The total sample consisted of six female (from five different mothers) and four male (from three different mothers) infants. The infants had no apparent visual, hearing or locomotor impairments or visible diseases.

For each subject, we screened, coded and analysed all four weeks of their second month and all four weeks of their ninth month of life. These two developmental points were chosen because they represent developmental milestones of capuchin monkeys (e.g. Verderane & Izar, 2019). The second month represents the beginning of the infants’ acquisition of physical and behavioural independence. Even though it is a very early stage, capuchin infants already engage with the environment and the other monkeys, showing some behaviours that are not present in the first month of life. In the ninth month, monkeys are still in their infancy phase and rely on their mothers; however, they are very active and possess quite a rich behavioural repertoire. They start becoming independent in the ninth month, when weaning usually starts (Verderane & Izar, 2019).

### 2.3. Field site and data collection

The study was conducted at Fazenda Boa Vista (9°39′ S, 45°25′ W), northeastern Brazil. Fazenda Boa Vista (1250 ha) is a private area located at the ecotone Cerrado–Caatinga. The climate is semi-arid with an average annual rainfall varying from 66.1 mm in the dry season up to 1011.3 mm during rainy season (Izar, 2017). The area is covered mainly with plain woodlands, predominantly medium-sized trees and palms (Verderane et al., 2020). There are also agricultural areas, such as fruit tree plantations and small corn fields (Spagnoletti et al., 2016).

We studied a group of capuchin monkeys designated as ‘Chicão’. This group has been studied since 2006 by PI and is habituated to the presence of people, especially the two field assistants (Marcos Fonseca de Oliveira and Arizomar da Silva Oliveira), who have been working with these animals since 2006. For the purpose of this study, we used footage from 2014–2018. During this period, the group ranged from nine individuals (one male alpha, one male subordinate, one female alpha, three female subordinates, two juveniles and one infant) to 16 individuals (one male alpha, two male subordinates, one female alpha, five female subordinates, five juveniles and two infants).

Since 2013, there has been a team dedicated to the data collection of social behaviours of the capuchin monkey infants of this group, from birth to 3 years of age. The field assistants accompany the group for 5 days a week, from dawn until dusk. In order to register the behaviour of each infant by a similar amount, two focal infants – randomly pulled – are observed per day, one in the morning and one in the afternoon. The order of infants varies one week to another and is organised in such a way that data collection becomes as homogenous as possible.

### 2.4. Procedures

#### 2.4.1. General procedure

First, NA conducted a thorough training with ACV and MB in using the software Noldus Observer XT 15.0 for video coding and behaviour identification. Taking into consideration the behaviours and behavioural categories of interest, the researchers were trained to identify events of lipsmacking or behavioural responses. Once training was completed, we conducted a reliability test between NA and the other researchers and reached concordance higher than 80%.

From that, we conducted a careful screening of the behaviours and behavioural categories of interest. For lipsmacking, 304 events were identified. Each event was defined as the opportunity of coding lipsmacking where both the beginning and ending of the behaviour could be observed. Each event was composed of a dynamic facial expression, which consisted of repeated, rhythmic and rapid opening and closing of the mouth (vertical movement), with or without tongue protrusion. A lipsmacking event may occur in isolation or in a sequence, which is determined by the temporal distance between one bout and another. These events were randomised and 219 (Acerola, 15; Michele, 7; Cenoura, 45; Duca, 30; Dançarina, 27; Peteca, 30; Oliveira, 7; Caititu, 22; Cacau, 24; Dourado, 12) were pulled from the dataset for coding. We used the maximum number of lipsmacking events for each individual, with the exception of those who had more than 30 events. For two of the three individuals with more than 30 events, we randomised the events and picked 30. For the last individual, who had many more events than the others, we chose to randomise and pick 45 events. This was due to (a) not wanting datasets that were too different from each other and (b) sampling effort, as we would not be able to code all of the events we had available. In the ninth month, there was only a record of 12 lipsmacking events; therefore, only the second month was analysed for the purpose of this study.

Further training took place, this time for coding. Once training was over, 10% of lipsmacking events were analysed for interobserver reliability by independent coders (Cohen's kappa). Interobserver reliability was excellent (≥0.8) for durations of the behaviours, which were the measures used for the analyses in this study. ACV and MB then proceeded to encode the events/videos that had been previously drawn. All coding and reliability tests were performed on Observer Noldus XT 15.0. We looked at the lipsmacking behaviour using real speed and frame-by-frame codification from two prisms: (a) the individual that is the target of the display (receiver, the infant); and (b) the individual that exhibits the display (emitter). For the receiver, we investigated the type of physical contact and the direction of their head. For the emitter, we investigated where lipsmacking was directed (the direction of the head of the emitter) and whether there was an active search for the infants’ face. Seeking the face was defined by behaviours such as moving the face towards the infant's face and accompanying the infant's face during the lipsmacking display. Moreover, we coded and analysed some characteristics of the display itself, i.e. associated facial expressions (tongue protrusion, tongue out, open mouth, scalp lifting). Direction of the head was devided into ‘directed to the face’, ‘directed to the head’, ‘directed to the hand’, ‘directed to other body parts’ and ‘not_emitter’ or ‘not_receiver’ (i.e. not directed at the individual with which the lipsmacking is occurring), and was determined by the direction of the animal's face (or the front of their head) in relation to the target area of the other animal in the interaction.

Emitters’ identity (mother, non-mother kin and non-mother non-kin), sex (female or male) and age, as well as the receivers’ age, sex and identity, were taken into consideration. A specific ethogram was created by the research team for this study (see Supplementary Materials).

### 2.5. Data analysis

We analysed a total of 128 videos (a total of 5,623.95 seconds) of naturalistic observations. There were 219 events analysed, with a minimum time of 0.14 s, a maximum time of 44.66 s, a mean of 3.168 and a standard deviation of 5.92.

First, we conducted a linear mixed model (LMM) to investigate potential effects on the time animals spent exhibiting lipsmacking. To analyse the first hypothesis, that lipsmacking is a face-to-face interaction, we first investigated to which parts of the infant's body the lipsmacking was directed (duration data): face, head, hand, other body parts, not_receiver. Moreover, when lipsmacking was directed towards the infants’ face, we analysed whether the emitter was actively seeking eye contact (duration data). Second, we coded the individual who received lipsmacking (infant) and investigated where they spent most of the time looking during the exhibition of the display (duration data): face, head, other body parts, not_emitter. We also analysed whether they spent most of their time in physical contact with the emitter. We analysed physical contact between receiver and emitter using the duration of the behaviours ‘touching with hand’, ‘grabbing members or tail’, ‘other physical contact’ and ‘no contact’. Another hypothesis was that lipsmacking is a behaviour that occurs mainly between mother and infant. We then conducted a descriptive analysis of the frequency with which the display occurs in mother–infant interactions, kin–infant interactions, or non-kin–infant interactions. Finally, to test the hypothesis that associated facial expressions (i.e. those that occur simultaneously to lipsmacking) regulate the duration of lipsmacking, we conducted a descriptive analysis and proceeded with a LMM. Total time of associated facial displays consists of the sum of the duration of tongue protrusion, scalp lifting, open mouth and tongue out.

To analyse lipsmacking duration, we applied a logarithmic transformation given its strong asymmetry. An LMM model with fixed factors *sex of receiver*, *sex of emitter* and *familiarity of emitter*, and random effects of *emitter identity* and *receiver identity* was used. Adjusted estimates with confidence intervals were presented to the final model. Model adjustments were evaluated by visual inspection of residuals.

To compare the duration of time the emitter was looking at the receiver's face, hand, head, not_receiver or other parts, during the lipsmacking event, we created an index to correct by the total lipsmacking duration, which varied for each event. Given a high percentage of 0 (59.5%) and 1 (4.1%), a non-parametrical approach was adopted using the Friedman test, that considers the within-event block. We used the Bonferroni correction for the post-hoc comparisons. A non-parametric effect size effect, analogous to Cohen's *d*, was presented and we considered a strong effect size when above 0.5, and a moderate effect size when between 0.3 and 0.5. Descriptive measures such as median, interquartile range, means and standard deviation are presented in the Supplementary Materials. The comparison of seeking and not seeking conditions was performed by the Wilcoxon ranked-signed test. All other comparisons of interest (allocation of time according to head direction of receiver and contact of receiver) were also analysed with a non-parametric approach owing to unworkable distribution (high percentage of 0 and 1).

To analyse the relationship between lipsmacking duration and associated facial expressions (total duration) of the emitter, a second LMM model was used with *associated facial expressions* as an explanatory variable, and random effects of *emitter identity* and *receiver identity*. The result of this model has an interpretation similar to a log-level regression, i.e. each 1 s increase in the explanatory variable (associated emotional expression's duration) causes a percentage increase in the dependent variable (lipsmacking duration), which was estimated using the equation. Model adjustments were evaluated by visual inspection of residuals.

All results were interpreted using a 5% significance level. The LMM models were performed using SAS University Edition (Statistical Analysis System) and all other analyses were performed in the software IBM SPSS 24. The ethogram used for behavioural codification is included in Supplementary Materials.

## 3. Results

From the 219 lipsmacking events, three were displayed by the mother of the infant, 61 by a non-mother related (kin) and 152 by a non-mother unrelated (non-kin) individual. In three other cases, it was not possible to identify the emitter.

We observed no significant effect of the sex of the receiver (F(1,133) = 0.38, *p* = 0.5363), the sex of the emitter (*F*(1,133) = 1.30, *p* = 0.2554) nor the familiarity of the emitter (*F*(1,133) = 1.04, *p* = 0.5947) on lipsmacking duration. Thus, the model with only the intercept and random effects of emitter identity and receiver identity was fitted to obtain the adjusted estimate of lipsmacking duration, which was on average 2.81 s (CI 95% = [2.1506; 3.6716]).

**Figure 1.**
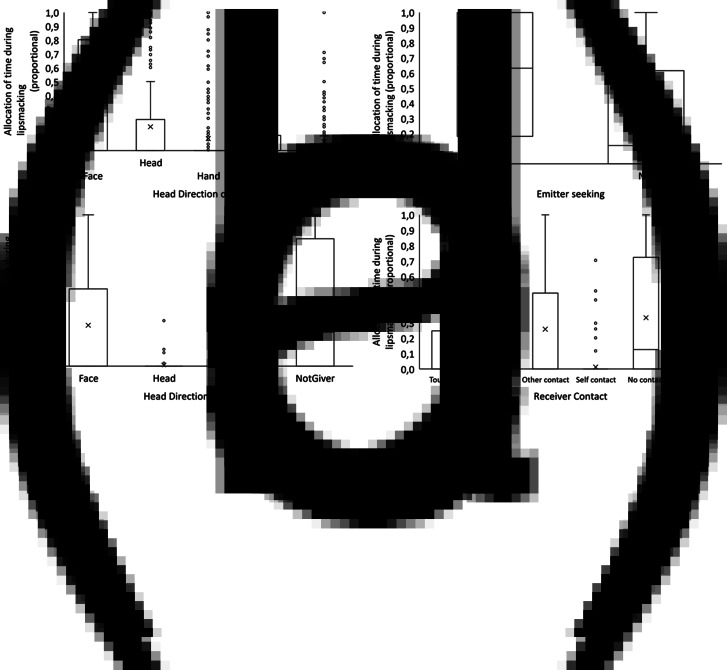
Box plot for allocation of lipsmacking duration: (a) for each head direction of the emitter; (b) with the emitter seeking or not visual contact with the infant; (c) for each head direction of the receiver; and (d) for each contact of the receiver.

Regarding the allocation of time during which the emitter directed their head to the infant during lipsmacking (Figure 1a), we found a significant difference between face, hand, head, other parts and not_receiver (Friedman *χ*^2^ = 109.5, d.f. = 4, *p* < 0.0001). The two-by-two comparisons corrected by Bonferroni indicated that emitters spent more time directed to the face of the infant during lipsmacking, compared with all other directions (*p* < 0.0001), and that they also spent more time directed at the head when compared with the hand. The comparison between the time allocated to hand and face resulted in a stronger effect size, greater than 0.5. All other effect sizes regarding comparisons with head direction were considered moderate (greater than 0.3). Regarding the allocation of time that the emitter spent seeking or not seeking visual contact with the infant during lipsmacking (Figure 1b), we found a significant difference: emitters spent more time seeking during the lipsmacking than not seeking (*Z* = −4.915, *p* < 0.0001). The effect size was considered moderate (greater than 0.3).

Regarding the allocation of time that receiver directed their head to the emitter during lipsmacking (Figure 1c), we found a significant difference between face, head, other parts and not_emitter (Friedman *χ*^2^ = 236.9, d.f. = 3, *p* < 0.0001). The two-by-two comparisons corrected by Bonferroni indicated that receivers spent more time, during lipsmacking, directed at not_emitter compared with the head or other parts (*p* < 0.0001), with a strong effect size. However, they also spent more time directed at the face when compared with the head (*p* < 0.0001) or other parts. The comparison between face and head presented a strong effect size, and the comparison between face and other parts was considered moderate. The comparison between face and not_emitter was not significant. Regarding the allocation of time related to contact by the receiver during lipsmacking (Figure 1d), we found a significant difference between behaviours (Friedman *χ*^2^ = 294.6, d.f. = 5, *p* < 0.0001). From Figure 1d, we observed that the receiver spent more time in no contact, other contact and touching, and a small amount of time in grabbing, self-contact and not_emitter. The two-by-two comparisons corrected by Bonferroni indicated that the receiver spent significantly more time, during lipsmacking, not doing any kind of contact when compared with grabbing, self-contact and not_emitter (*p* < 0.001, with strong effect size) and when compared with touching (*p* = 0.003, however with a weak size effect). Similarly, the receiver also spent significantly more time doing other contact (e.g. head touching back) when compared with grabbing, self-contact and not_emitter (*p* < 0.001, with a moderate size for the first comparison and strong effect size for the last two comparisons). Finally, the receiver spent significantly more time, during lipsmacking, touching when compared with grabbing, self-contact and not emitter (*p* < 0.001, with a moderate effect size for all these three comparisons). The comparison of no contact and other contact was not significant.

Furthermore, we looked at 94 events of lipsmacking to investigate whether associated facial expressions, such as scalp lifting, tongue out, open mouth and tongue protrusion, could have an effect on the display of lipsmacking. We observed that the total duration of associated facial expressions was associated with the lipsmacking duration (*F*(1,60) = 24.46, *p* < 0.0001), for each 1 s increase in total duration of emotional expression, there was an estimated increase of 32.59% (CI 95% = [19.4; 45.8]) in total duration of lipsmacking.

## 4. Discussion

In this study, we advance the literature on the structure of lipsmacking behaviour, by conducting a systematic work in terms of a behavioural cue that is associated with socio-emotional regulation. Here, we investigated free wild animals of a population of capuchin monkeys from Brazil, which allowed the analysis of the natural spontaneous behaviour of the animals. Moreover, because data collection resulted in video recordings of focal animal observations of infant capuchin monkeys, we were able to investigate individuals at very young ages (2 months old) and to analyse subtle behaviours, such as gaze direction.

We show that duration of lipsmacking was not affected by the sex of the receiver, the sex of the emitter nor the familiarity of the emitter. Moreover, emitters spent more time looking at the infant's face, compared with other parts of the infant's body and to other individuals that were not the receiver, and more time seeking eye contact from the receiver than not seeking. On the other hand, receivers spent more time looking away from the emitter (not_emitter), compared with the head and other parts of the emitter's body, and more time looking at the face of the emitter, compared with the head and other parts of the emitter's body. Interestingly, there was no significant difference between face and not_emitter. In fact, the average relative time looking away from the emitter was higher (0.412±0.411) than the average relative time looking at the emitter's face (0.267±0.347). We also found that infants spent more time in no contact and ‘other contact’, such as head touching belly or back touching back. There was no significant statistical difference between no contact and other contact. Finally, lipsmacking did not occur only with the mother and other facial expressions exhibited at the same time as the display influenced the maintenance of the lipsmacking behaviour (for every 1 s increase in duration of associated facial expressions there was an increase of 32.59% in lipsmacking duration).

In mammals, the beginning of life of each individual is very sensitive and, thus, interactions with partners might be critical for the development of their social abilities (Thompson & Cords, 2019). In fact, in this study, for the infants drawn, from the 300+ events of lipsmacking in the second month, we found only 12 lipsmacking events in the ninth month, which were not considered in the analyses owing to its low number. In Verderane et al. (2020), lipsmacking in capuchin monkeys occurred in higher frequencies in the second month, with a strong fall in the third month and a second, although more subtle, rise in the ninth month. However, this may be due to the framework of the paper, the purpose of which was to assess only mother–infant behaviour. In our study, we investigated whether the sex of the receiver, the sex of the emitter and the familiarity of the emitter had an effect on the duration of lipsmacking. Age was not considered as a factor since all of our analysable cases occurred in the same time frame (second month). We found none of these factors impacted the exhibition of lipsmacking, which means that the emission and reception of the display were not dependent on sex and that the familiarity of emitter (mother, non-mother kin, non-mother non-kin) did not affect the structure of the display. In Ferrari et al. (2009) the frequency of the display increased with age and they found a significant interaction between age and familiarity of the partner. There was a drastic decrease in the exhibition of lipsmacking after the first month of life, which could be due to infants’ physical development (e.g. separating from their mother) and their psychological development (e.g. interest in same-age conspecifics). These different results might be due to differences in the species studied.

Our data show that the time emitters spent looking at the infant's face was greater than the time emitters spent directed at the infant's head, hands, other body parts and away from the receiver. At the same time, by analysing when lipsmacking occurred towards the face of the receiver, we found a significant difference between seeking and not seeking the face, meaning that animals who performed lipsmacking towards the infant's face did so by actively seeking eye contact with the infant. However, the time receivers spent looking away from the emitter and the time they spent looking at the face of the emitter were greater than the time directed at head and other body parts. Looking more in depth, infants did spend a great deal of time (37%) looking at the face of the emitter but spent even more time (57%) looking away. Even though this last comparison was not statistically different, it is still important to look at the descriptives. According to Gallo et al. (2022), to make visual information exchange effective, both parties must be attentive to the face of the other, so a correct and successful decoding and responding can occur (Gallo et al., 2022). In fact, an attention bias to positive stimuli, such as a happy face, can play a critical role in early socio-emotional functioning and processing (Rayson et al., 2021). However, our findings in combination bring into question the idea that lipsmacking is solely a face-to-face interaction (e.g. Verderane et al., 2020) or is reliant on mutual gaze (Ferrari et al., 2009). We suggest that this display might have an affiliative function even when it does not encompass eye-to-eye contact and further studies must investigate behavioural changes when lipsmacking is received with mutual gaze compared with when it is not. An alternative explanation is that other mechanisms come into place when the display is not exhibited in face-to-face interactions. Future studies must look into other possible functions.

Moreover, from the receivers’ perspective, we aimed to examine whether they would be in physical contact with the emitter during the exhibition of the display. We found that ‘no contact’ showed higher means in all of the comparisons (touching with hand, grabbing members or tail, self-contact and not_emitter), with the exception of what we called ‘other physical contact’, which includes, for instance, being in such close proximity that the bodies are in contact. ‘Other contact’ was also longer than grabbing, self-contact and not_emitter. This means that when lipsmacking occured with infant capuchin monkeys, we observed physical contact such as ‘head touching body’ more often than the expected ones, such as touching with the hand. In the study of Ferrari et al. (2009) with rhesus macaques, they found two patterns of lipsmacking involving high rates of active physical contact, one where the mother held the infant's head and pulled it towards her face and another where the mother separated the infant from the rest of the group and actively sought the infant's face, by bouncing and lowering her head. In their study, around 22% of lipsmacking events occurred in ventral–ventral contact (Ferrari et al., 2009). However important, these results illuminate the type of physical contact that emitters have with infants during lipsmacking. In our case, we contribute with data on the type of contact receivers have with emitters. Our results are initial and suggest that more in-depth studies must be conducted to clarify how lipsmacking occurs.

Lipsmacking is a display involved in socio-emotional regulation. However, both for the emitter and the receiver of the behaviour, there are important aspects that have not been addressed yet. From our investigation with capuchin monkeys, animals not often studied in terms of socio-emotional regulation, lipsmacking occurred between infants and a variety of animals within the group: other infants, juveniles and adults. In fact, most of the lipsmacking events we targeted occurred with non-mother non-kin (different matrilines) individuals (*n* = 152), then with non-mother related individuals (*n* = 61), then with the mother (*n* = 3). This is not in line with most of the literature, that shows that lipsmacking occurs mainly with the mother, possibly because in capuchin monkeys the infants are transported and carried on the back of the individuals. Verderane et al. (2020) discuss that lipsmacking is a mother–infant interaction and describes the occurrence of the behaviour in different points of the development of capuchin monkeys, but only between mothers and their infants. Here, we show that lipsmacking is exhibited much more with other individuals that are not the mother. In fact, by using another prism when investigating lipsmacking (i.e. considering that the display occurs between infants and non-mother individuals), researchers may find more lipsmacking occurrences in their own data. De Marco and Visalberghi (2006) show that face-to-face behaviour related to the exhibition of facial displays amongst infant capuchins is observed more often with peers, less with adults and almost never with the mother. In 2015, Fedurek et al. found that chimpanzees emitted lipsmacking when grooming vulnerable parts of the receiver's body and often produced this signal when premature termination of the interaction was highly probable. However, this behaviour may be exhibited between individuals of different ages and levels of hierarchy and is not related to well-affiliated or higher-ranking individuals (Fedurek et al., 2015). These results are probably closer to ours. On the other hand, Ferrari et al. (2009) investigated mother–infant rhesus macaque dyads during the first two months of the infant's life in a captive setting. Infants received more lipsmacking by their mothers than from other individuals. Moreover, the frequency of the display increased with age and they found a significant interaction between age and familiarity of the partner. Thus, even though lipsmacking can be a behaviour used by mothers to interact with their offspring, our data show that for capuchin monkeys the social and emotional regulation mechanisms that are involved in the production, exhibition and reception of lipsmacking relate more to other individuals.

When we looked at the associated facial expressions that are exhibited simultaneously with lipsmacking, i.e. scalp lifting, open mouth, tongue out and tongue protrusion, we found that for each 1 s increase in the duration of associated facial expressions, there was an increase of more than 30% in lipsmacking duration. This shows that the presence of these associated expressions worked on the maintenance of lipsmacking. According to Gallo et al. (2022), one typical context-dependent signal that primates use is the relaxed open mouth, or the so-called ‘play face’. The play face is used to express positive emotions and serves as an anticipation of the affiliative nature of some behaviours. It is possible that the associated facial expressions, such as open mouth, function as signals to anticipate affiliative interactions and are used to enhance the communicative value of lipsmacking. Fedurek et al. (2015) looked at how lipsmacking facilitates the expression of grooming. In our case, we approached the topic from a different but complementary direction: what facilitates the exhibition of lipsmacking towards an infant and what helps the maintenance of such kind of interaction. Associated facial expressions may facilitate lipsmacking while lipsmacking facilitates social behaviour.

According to Morrill et al. (2012), lipsmacking is an affiliative signal observed in many non-human primate species. Most importantly, lipsmacking is one of the first facial expressions produced by infant monkeys (De Marco & Visalberghi, 2007; Ghazanfar et al., 2012). Emotional regulation is central to the occurrence of affiliative behaviours since they require one individual to express their sensations and motivations and another individual to perceive the emotional expression of others, thus, animals must regulate their emotional experience. For this emotional regulation to occur, signals, such as the lipsmacking behaviour, are produced for emotional information exchange. Therefore, when we speak about lipsmacking at a social level, we are intrinsically speaking of socio-emotional mechanisms. In fact, Maestripieri (1997), suggests that this facial gesture is likely to carry the most communicative meaning for non-human primates. This means that we are looking at a socially relevant behaviour that is linked to emotional expression and perception and is already present at very young ages.

In primate evolution, there was a tendency towards the increase of the size and complexity of social groups when individuals became more sensitive to social visual cues, such as facial expressions, for communication (Parr et al., 2000). In fact, primates are known to produce facial expressions in a greater variety and frequency than other groups of animals (Micheletta et al., 2012). At the same time, non-human primates are known to be able to decode information from visual and acoustic emotional displays (Ghazanfar & Logothetis, 2003). In fact, emotional processes are central to the exhibition of affiliative behaviours and to the regulation of social interactions. Emotions drive the behaviour of organisms and will provide individuals with the tools to interact with their world. In fact, Ferrari et al. (2009) believe that the function of lipsmacking is allowing communicative exchanges that promote opportunities of emotional development. Lipsmacking is such an important display in non-human primates that Kavanagh et al. (2022) believe that while there is no evidence of a direct counterpart in humans, it is possible that future data may identify continuity with this expression in people.

## 5. Conclusion

Our findings suggest that emitters are very engaged with the infant during lipsmacking, looking longer towards the receiver's face than to other parts of their body and seeking eye contact during the presentation of the stimulus. On the other hand, infants do not do everything they can as receivers of this display. For instance, they spend as much time looking away from the emitter as looking at the emitter's face and they spend most time in no contact or in other contact than touching and grabbing the emitter. It is possible that these animals have evolved strategies to attract the infant's attention, but the behaviour occurs regardless. According to Micheletta et al. (2012), facial expressions are usually studied as static and invariant sets of components, or each component is studied in isolation. However, even subtle dynamic changes in the facial display can be meaningful to the receiver of the message. It may also be the case that infants do not need to be looking at the face of the emitter for the whole presentation of the stimulus. Maybe seeing the display for a fraction of its total duration is sufficient for emotional exchange.

Even though there is evidence showing that lipsmacking is a mother–infant face-to-face interaction, our findings suggest there may be other mechanisms in place when it comes to this socio-emotional display. Lipsmacking is not solely a mother–infant interaction, with results pointing to this behaviour being frequently exhibited between infants and non-mother individuals. We also found that accompanying lipsmacking with facial expressions such as scalp lifting, open mouth, tongue out and tongue protrusion will facilitate the exhibition of the display and may act as a key factor for the maintenance of the behaviour. Also important, the duration of lipsmacking in capuchin monkeys is not affected by intrinsic variables such as the sex of the receiver, the sex of the emitter and the familiarity of the emitter.

Lipsmacking, which involves dynamic facial movements, eye and body contact, might be one of the most important signals expressed by non-human primates (Maestripierei, 1997). Studies on the function and evolution of facial expressions improve our understanding of the evolution of broader systems, such as communication. Further, they provide new frameworks to analyse social interactions in a more general way (Micheletta et al., 2012). Investigating lipsmacking in wild animals that are reared on the back of their mothers and, thus, have the opportunity to interact with a greater variety of individuals, might be key for the understanding of socio-emotional regulation mechanisms in capuchins, neotropical monkeys and primates in a broader sense.

## Supplementary material

For supplementary material accompanying this paper visit https://doi.org/10.1017/ehs.2023.10. click here to view supplementary material

## Research transparency and reproducibility

The complete ethogram and a table of the descriptive measures related to the total lipsmacking duration are available in the online Supplementary Information. The dataset used for this study is available from the corresponding author upon request.

## Data accessibility

Data are available from the corresponding author upon request.
