# The shape of lipsmacking: socio-emotional regulation in bearded capuchin monkeys (Sapajus libidinosus) – CORRIGENDUM

**DOI:** 10.1017/ehs.2024.4

**Published:** 2024-03-04

**Authors:** Natalia Albuquerque, Carine Savalli, Marina Belli, Ana Clara Varella, Beatriz Felício, Juliana França, Patrícia Izar

The authors apologise for some text errors detailed below:

Page 2, Line 50 ‘Verderane et al. (2020) investigated capuchin monkeys (*Sapajus libidinosus*) in Brazil…’ should read ‘Verderane et al. (2020) investigated the same population of capuchin monkeys *(Sapajus libidinosus*) as the present study, in Brazil since both studies were conducted using the same database’.

The text implies that Verderane et al concluded that lipsmacking was solely a part of face-to-face interaction. Verderane et al did not assess the breadth of contexts in which lipsmacking may occur, they investigated lipsmacking in the context of face-to-face interactions of mother-infant interaction only, therefore:

Page 3, Line 1 ‘They found that lip-smacking was a face to face behaviour’ should read ‘They investigated lip-smacking as an element of mother-infant face-to-face interactions'.

Page 10, Line 7 ‘Verderane et al (2020) discuss that lipsmacking is a mother–infant interaction.’ should read ‘Verderane et al (2020) discuss that lipsmacking is an element of mother–infant interaction.’

The description, accessibility, and ownership of the dataset is not complete, therefore:

The following text should be added at the end of Page 4, Section 2.3 ‘the data on which this study is based form part of the larger LEDIS dataset, owned and managed by Patrícia Izar, Department of Psychology, University of São Paulo. The videos annotated for the research in this article form part of a broader video library, also used for other research projects and available upon request to ledsapajus.ip@usp.br.’
