# Supplementary material for: The shape of lipsmacking: socio-emotional regulation in bearded capuchin monkeys (Sapajus libidinosus)
Source: Evol Hum Sci. 2023 May 12;5:e16. doi: 10.1017/ehs.2023.10 (PMC10426065; doi:10.1017/ehs.2023.10)

**Have you ever heard of Lipsmacking?**

Lipsmacking behaviour is a rhythmic facial expression (rapid closing and opening of the mouth) observed in many non-human primate species. It is an affiliative signal, which means that it sends a positive message and promotes affiliative interactions. Emotional regulation is central to the occurrence of affiliative behaviours since they require one individual to express their sensations and motivations and another individual to perceive the emotions of the first. Animals must regulate their emotional experience. For this emotional regulation to occur, signals, such as the lipsmacking behaviour, are produced for the exchange of emotional information. In capuchin monkeys, lipsmacking is usually directed at infants.


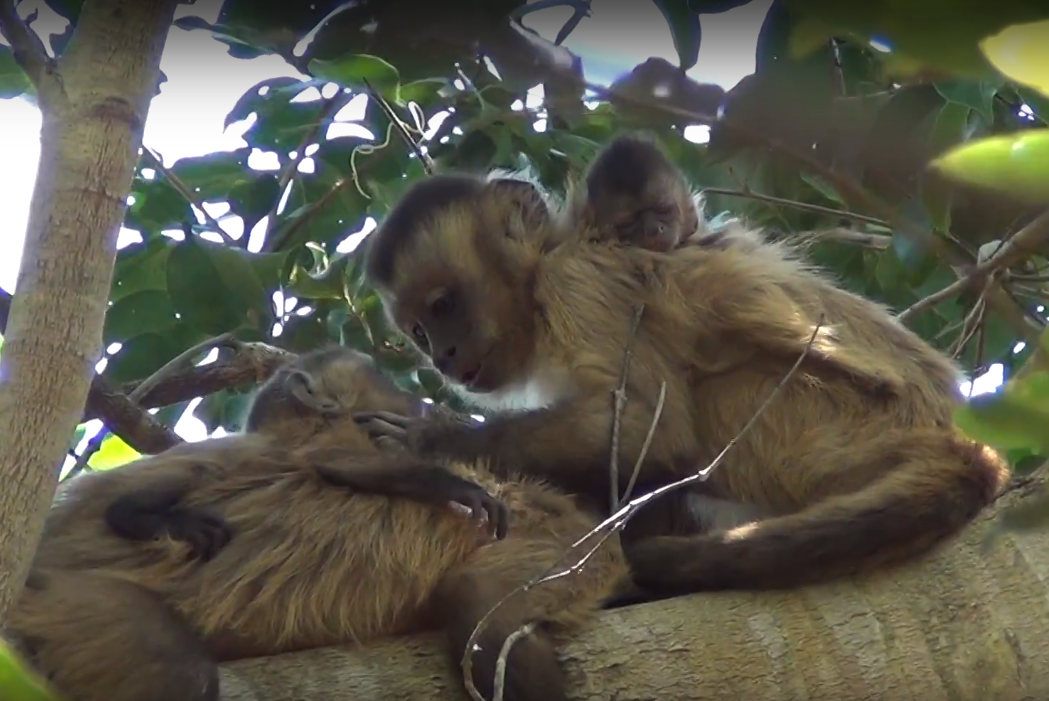


Source: LEDIS - USP

In a recent study, Albuquerque and colleagues (2023) conducted in-depth investigations of wild infant capuchin monkeys (*Sapajus libidinosus*) of Fazenda Boa Vista (Piaui, Brazil) concerning lipsmacking to explore the structural aspects of this behaviour. They investigated a group of capuchin monkeys designated as “Chicão”, which has been studied since 2006 by Professor Patrícia Izar (co-author of the paper) and is habituated to the presence of people. For this paper, Albuquerque and colleagues analysed a total of 128 videos (total of 5,623.95 seconds) of naturalistic observations and coded 219 events of lipsmacking using real time and frame-by-frame speed.

Their first aim was to examine whether the duration of lipsmacking would be affected by intrinsic variables. They found that duration of lipsmacking was not affected by the sex of the receiver, sex of the emitter nor familiarity of the emitter.

They also tested the hypothesis that lipsmacking is a face-to-face behaviour. They predicted that (i) the time infants spend looking at the emitter’s (who produces lipsmacking) face is higher than looking at other parts of the emitter’s body; (ii) the time infants spend in physical contact with the individual who is displaying the expression is greater than the time infants spend in no contact; (iii) the time emitters spend looking at the infant’s face is higher than looking at other body parts; and (iv) the time emitters spend actively seeking the face of the baby is greater than the time they spend in no active search of the infant’s face. They found that emitters did spend more time looking at the infant’s face, and more time seeking eye contact from the receiver than not seeking. However, receivers spent a similar amount of time looking at the emitter’s face and away from the emitter. Moreover, infants spent a similar amount of time in no contact and “other contact” (e.g. head touching belly or back touching back).


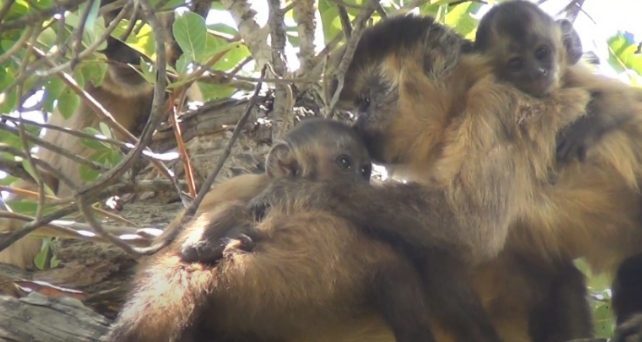


Source: LEDIS - USP

Their second hypothesis was that lipsmacking is a behaviour that occurs between mother and infant. However, they found that from all lipsmacking events analysed, 1,5% was displayed by the mother of the infant, 28,5% were displayed by a non-mother related individual (kin) and 70% by a non-mother unrelated individual (non-kin). They also looked at what they call “associated facial expressions”, which are expressions displayed concurrently to lipsmacking. They aimed to test the hypothesis that these associated expressions play a role in the occurrence of lipsmacking and the “lipsmacking interaction” between emitter and receiver, since redundant emotional information may boost discriminatory processes. They observed that the total duration of associated emotional expressions was associated with the lipsmacking duration: for each 1 second increase in total duration of emotional expression, there was an increase of 32.59% in total duration of lipsmacking.

These findings suggest that emitters are very engaged with the infant during lipsmacking, looking longer towards the receiver’s face than to other parts of their body and seeking eye contact during the presentation of the stimulus. On the other hand, infants do not do everything they can as receivers of this display. For instance, they spend as much time looking away from the emitter as looking at the emitter’s face and they spend most time in no contact or in other contact than touching and grabbing the emitter. It is possible that these animals have evolved strategies to attract the infant’s attention, but the behaviour occurs regardless. It may also be the case that infants do not need to be looking at the face of the emitter for the whole presentation of the stimulus. Maybe seeing the display for a fraction of its total duration is sufficient for emotional exchange. Moreover, even though there is evidence showing lipsmacking is a mother-infant face-to-face interaction, Albuquerque and colleagues’ findings suggest there may be other mechanisms in place when it comes to this socio-emotional display. They also found that accompanying lipsmacking with facial expressions such as scalp lifting, open mouth, tongue out and tongue protrusion will facilitate the exhibition of the display and may act as a key factor for the maintenance of the behaviour.


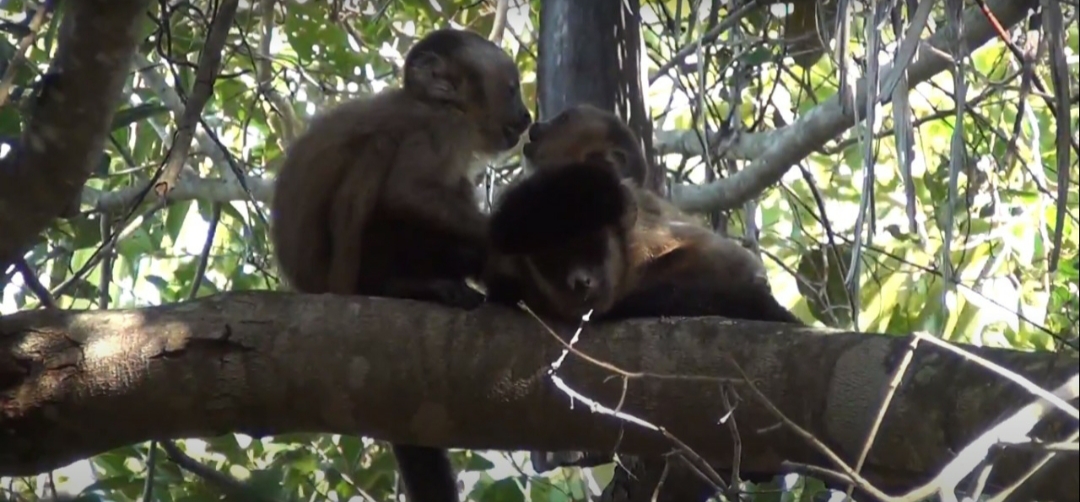


Source: LEDIS - USP

Studies on the function and evolution of facial expressions improve our understanding of the evolution of broader systems, such as communication. Further, they provide new frameworks to analyse social interactions in a more general way. Investigating lipsmacking in wild animals that are reared on the back of their mothers and, thus, have the opportunity to interact with a greater variety of individuals, might be key for the understanding of socio-emotional regulation mechanisms in capuchins, neotropical monkeys and primates in a broader sense.

*Natalia Albuquerque is a biologist, with a Masters and a PhD in Experimental Psychology - emphasis on Ethology (Animal Behaviour and Cognition). She has conducted a postdoctoral research on socio-emotional regulation in capuchin monkeys and is now a postdoc fellow of the University of São Paulo (Brazil) working on the expression of emotions in dogs.*


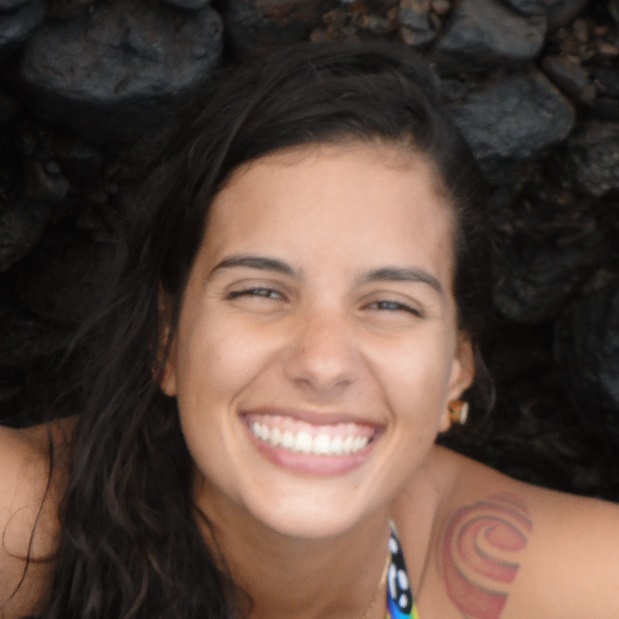

Supplement: Supplementary file 1 [file S2513843X23000105sup.zip › S2513843X23000105sup001.docx]
