# Supplementary material for: The shape of lipsmacking: socio-emotional regulation in bearded capuchin monkeys (Sapajus libidinosus)
Source: Evol Hum Sci. 2023 May 12;5:e16. doi: 10.1017/ehs.2023.10 (PMC10426065; doi:10.1017/ehs.2023.10)
Supplement: Supplementary file 1 [file S2513843X23000105sup.zip › S2513843X23000105sup003.docx]

SUPPLEMENTARY MATERIALS

**Title: The shape of lipsmacking: socio-emotional regulation in bearded capuchin monkeys (*Sapajus libidinosus*)**

**Authors:** Natalia Albuquerque¹*, Carine Savalli², Marina Belli¹, Ana Clara Varella¹, Beatriz Felício¹, Juliana França¹, Patrícia Izar¹

**Affiliations:** ¹Institute of Psychology, University of São Paulo, Brazil; ²Federal University of São Paulo, Brazil

*Correspondence to: nsalbuquerque@gmail.com

Table 1. Complete ethogram

| **BEHAVIOURAL CATEGORY** | **BEHAVIOUR** | **DESCRIPTION** |  |
| --- | --- | --- | --- |
| Lipsmacking | Lipsmacking | Dynamic facial expression, consisting of rapid, repeated, and rhythmic vertical movements of opening and closing of the mouth, with or without tongue protrusion. Usually, it is directed towards another individual, who might be an adult, subadult, juvenile or infant, and it can occur in isolation or in sequence. Lipsmacking is only recorded when both beginning and ending are identifiable. |  |
|  |  |  |  |
|  |  |  |  |
|  |  |  |  |
|  |  |  |  |
|  |  |  |  |
|  |  |  |  |
|  |  |  |  |
| Direction of the Head | Face | Face of focal individual is directed towards the face of the receiver/emitter of lipsmacking. If visible, gaze direction will be used to determine "direction of the head". |  |
|  |  |  |  |
|  | Head | Face of focal individual is directed towards the head of the receiver/emitter of lipsmacking. If visible, gaze direction will be used to determine "direction of the head". "Head" is considered as any part of the individual's body above the neck, with exception to the face. |  |
|  |  |  |  |
|  | Stomach | Face of focal individual is directed towards the stomach of the receiver/emitter of lipsmacking. If visible, gaze direction will be used to determine "direction of the head". "Stomach" is considered as the entire ventral area below the neck and above genitals, with exception to the superior members. |  |
|  |  |  |  |
|  | Genitals | Face of focal individual is directed towards the genitals of the receiver/emitter of lipsmacking. If visible, gaze direction will be used to determine "direction of the head". |  |
|  |  |  |  |
|  | Other parts | Face of focal individual is directed towards other parts of the body of the receiver/emitter of lipsmacking, that not the face, head, stomach or genitals. If visible, gaze direction will be used to determine "direction of the head". |  |
|  |  |  |  |
|  | Non-individual | Face of focal individual is directed towards anything that is not a visible individual. If visible, gaze direction will be used to determine "direction of the head". |  |
|  |  |  |  |
|  | Other individual | Face of focal individual is directed towards another individual, who is not involved in lipsmacking. If visible, gaze direction will be used to determine "direction of the head". |  |
|  |  |  |  |
|  | Covered head | Head of the focal individual is not visible, or direction of the head is not deductible from the video. |  |
|  |  |  |  |
| Facial Expressions | Facial display | Differential moving of the face potentially linked to the exhibition of affective states (e.g. eyebrow movements). Facial expressions will be recorded when at least one eye and the mouth of the individual are visible. |  |
|  |  |  |  |
|  | Tongue protrusion | Partial or full exposition of the tongue, when in direct contact with the upper and lower lips. |  |
|  |  |  |  |
|  | Tongue out | Full exposition of the tongue concurrently to the opening of the jaw. |  |
|  |  |  |  |
|  | Open mouth | Full opening of the jaw when there is no tongue out. |  |
|  |  |  |  |
|  | Neutral face | Absence of facial displays. |  |
|  |  |  |  |
|  | Covered face | When the criterion for the record of facial expressions is not met, i.e. mouth and at least one eye visible. |  |
|  |  |  |  |
| Visual Contact | Seeking | Active search for the visualisation of the face and/or gaze of another individual. This active search is measured by the moving of the head of the individual who seeks visual contact with the face of the other. The focal infant may be the actor or the receptor. |  |
|  |  |  |  |
|  | Not seeking | Visual access to the face of another individual, however with no active search for the visualisation of the face or the gaze of the other. The focal infant may be the actor or the receptor. |  |
|  |  |  |  |
|  | Not visible | The record of seeking or not seeking cannot be done because the moving of the head of the individual is not identifiable. |  |
|  |  |  |  |
| Physical Contact | Grabbing tail or members | An individual uses the fingers of one or two hands to hold the tail, posterior or inferior members of another individual. The focal individual may be the actor or the receptor. |  |
|  |  |  |  |
|  | Touching with hand | An individual touches with one or two hands, without grabbing, any part of the body of another individual. The focal infant may be the actor or the receptor. |  |
|  |  |  |  |
|  | Other physical contact | Any other form of physical contact with any part of the body of another individual with exception to grabbing and touching. |  |
|  |  |  |  |
|  | Scratching | Self-directed behaviour, involving repeated movements of the fingers or toes against the individual's skin. |  |
|  |  |  |  |
|  | Self-grooming | Self-directed behaviour, involving the inspection or manipulation of the individual's body surface. It may be performed with the hands, mouth or hands and mouth concurrently. |  |
|  |  |  |  |
|  | Absent | There is no type of physical contact in any part of the individual's body. |  |
|  |  |  |  |
|  | Not visible | When the occurrence of physical contact cannot be determined from the video. |  |
|  |  |  |  |

Table 2. Descriptive measures related to the total lipsmacking duration (219 events of lipsmacking).

| **Behavior** | **Condition** | **Median** | **Quartile range** | **Mean** | **Standard deviation** |
| --- | --- | --- | --- | --- | --- |
| **Seeking behavior of Emitter** | No | 0.121 | 0.615 | 0.317 | 0.370 |
|  | Yes | 0.632 | 0.818 | 0.579 | 0.395 |
| **Head Direction of Emitter** | Face | 0.303 | 0.802 | 0.403 | 0.393 |
|  | Hand | 0.000 | 0.000 | 0.087 | 0.212 |
|  | Head | 0.000 | 0.225 | 0.172 | 0.294 |
|  | NotReceiver | 0.000 | 0.046 | 0.066 | 0.157 |
|  | Other parts | 0.000 | 0.110 | 0.137 | 0.276 |
| **Head Direction of Receiver** | Face | 0.022 | 0.513 | 0.267 | 0.347 |
|  | Head | 0.000 | 0.000 | 0.010 | 0.074 |
|  | Other parts | 0.000 | 0.000 | 0.040 | 0.137 |
|  | NotGiver | 0.286 | 0.911 | 0.412 | 0.411 |
| **Contact of Receiver** | Grabbing | 0.000 | 0.000 | 0.060 | 0.216 |
|  | No contact | 0.126 | 0.724 | 0.332 | 0.389 |
|  | NotGiver | 0.000 | 0.000 | 0.032 | 0.159 |
|  | Other contact | 0.000 | 0.492 | 0.258 | 0.348 |
|  | Self-contact | 0.000 | 0.000 | 0.013 | 0.074 |
|  | Touching | 0.000 | 0.247 | 0.183 | 0.318 |
